# Supplementary material for: OnTAD: hierarchical domain structure reveals the divergence of activity among TADs and boundaries
Source: Genome Biol. 2019 Dec 18;20:282. doi: 10.1186/s13059-019-1893-y (PMC6918570; doi:10.1186/s13059-019-1893-y)
Supplement: Supplementary file 1 — Additional file 1. Supplementary figures and tables. [file 13059_2019_1893_MOESM1_ESM.docx]

|  |
| --- |

**Figure S1** | **Illustration of convoluted TAD structures. a,** Candidate TADs (a,c) and (b,d) are both suboptimal, as their scores may be driven by a real TAD (b,c). **b,** Two real TADs (a,c) and (b,c) are nested, which makes the score of (a,c) convoluted with the score of (b,c). **c,** Real TADs (a,c) and (b,d) are partially overlapping, which may be recaptured as nested TADs (b,c), (a,c) and (a,d).

| 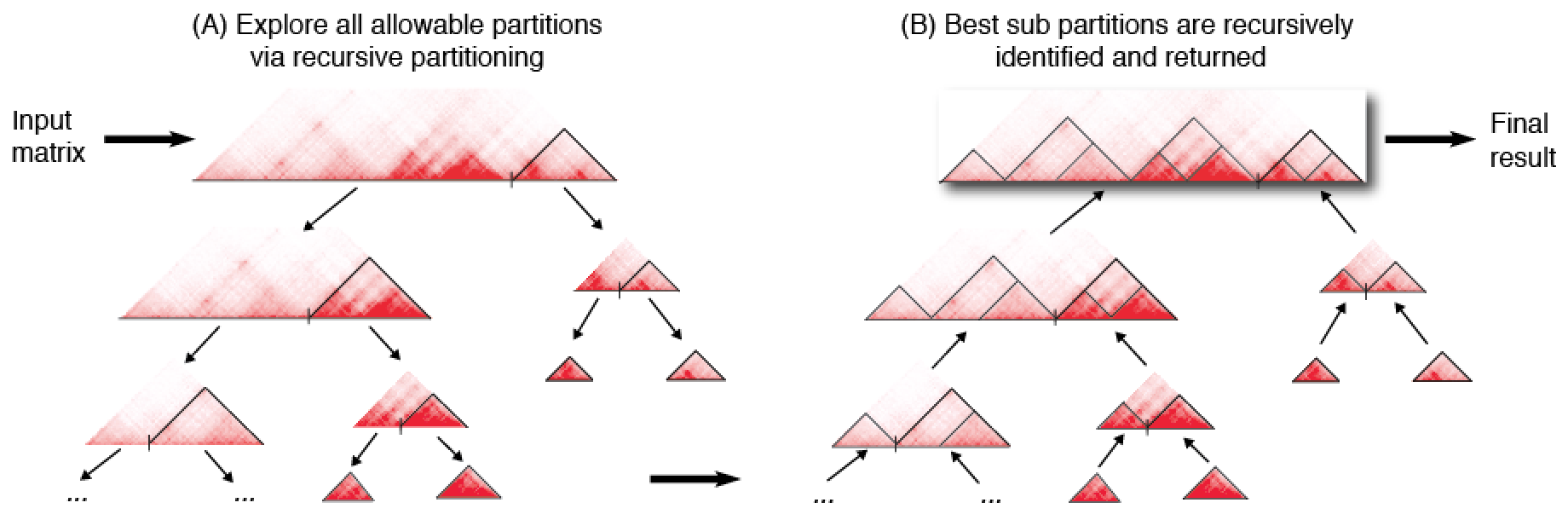 |
| --- |

**Figure S2** | **Illustration of the recursive TAD calling algorithm. a,** At the first step of the algorithm, the entire Hi-C matrix is partitioned into two matrices, the one forming the largest right-most TAD (i.e. triangles marked in black) and the remaining part, according to a score function. Then the same function is called on each sub-matrix to recursively identify nested TAD structures. **b,** Each recursion step identifies the best set of TADs in its matrix under consideration according to the score function, and returns the TAD calls back to its parent until the root.

| 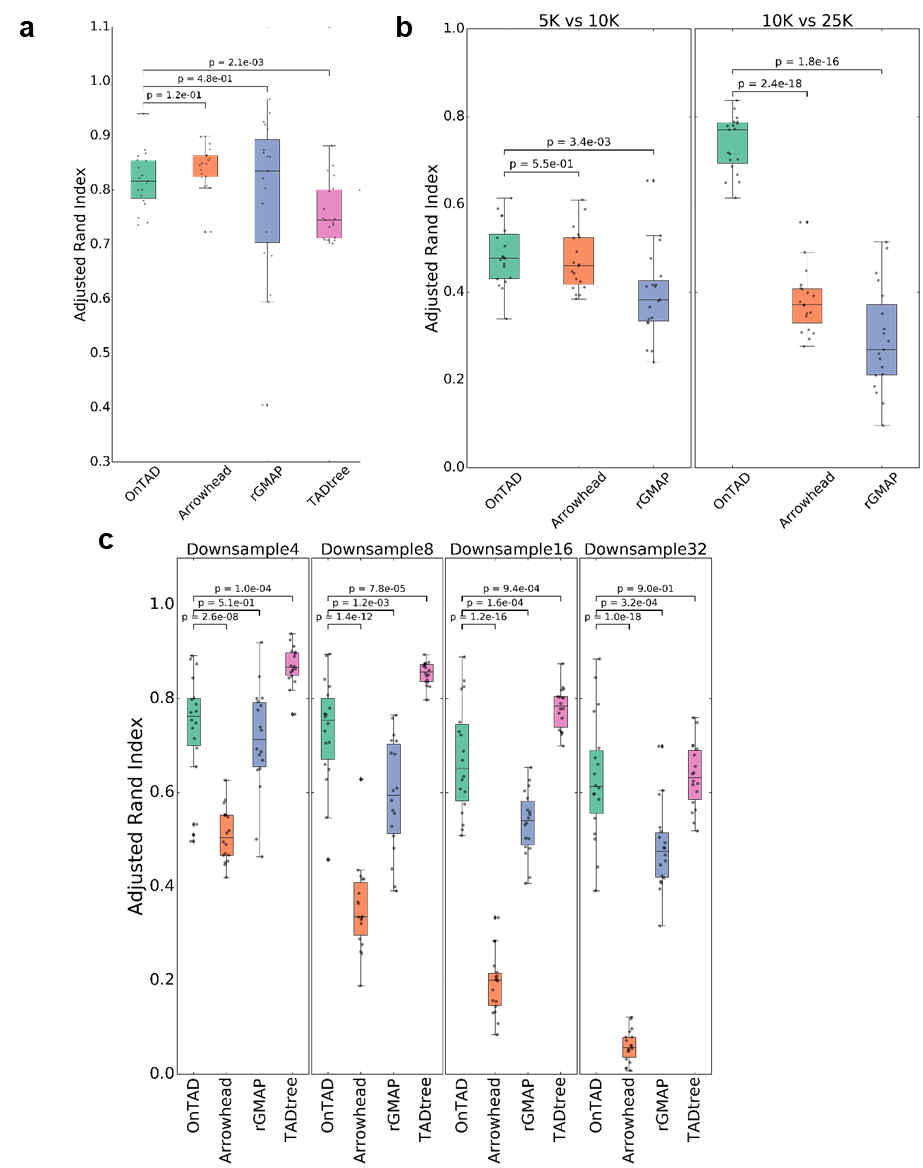 |
| --- |

**Figure S3 | TAD reproducibility under different measurements. a,** Adjusted rand index between TADs from two biological replicates (GM12878, 10Kb). **b**, Adjusted rand index across TADs from Hi-C data in multiple resolutions (GM12878, 5Kb, 10Kb and 25Kb). TADtree is not included because it has difficulty finishing the computation on high resolution data due to its high memory consumption. **c**, Adjusted rand index between TADs from Hi-C data in original sequencing depth and in different down sampled sequencing depth (GM12878, 1/4, 1/8, 1/16 and 1/32 of original sequencing depth). All p-values are calculated based on two-sided t-test.

| 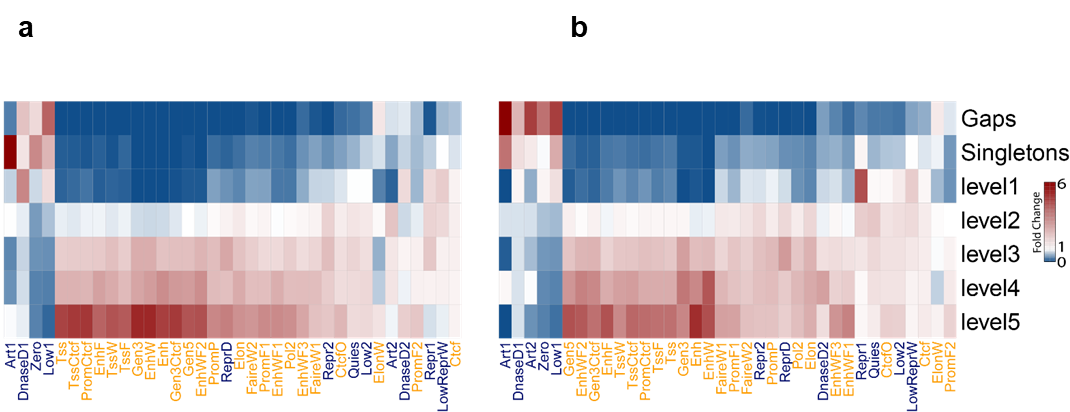 |
| --- |

**Figure S4** | **Enrichment of epigenetic states at the boundaries of different levels of TADs.** Enrichment of epigenetic states at the regions covered by different levels of TADs. The enrichment (fold change) of active states (orange states) increases as the TAD level increases. **a,** K562 **b,** Huvec

| 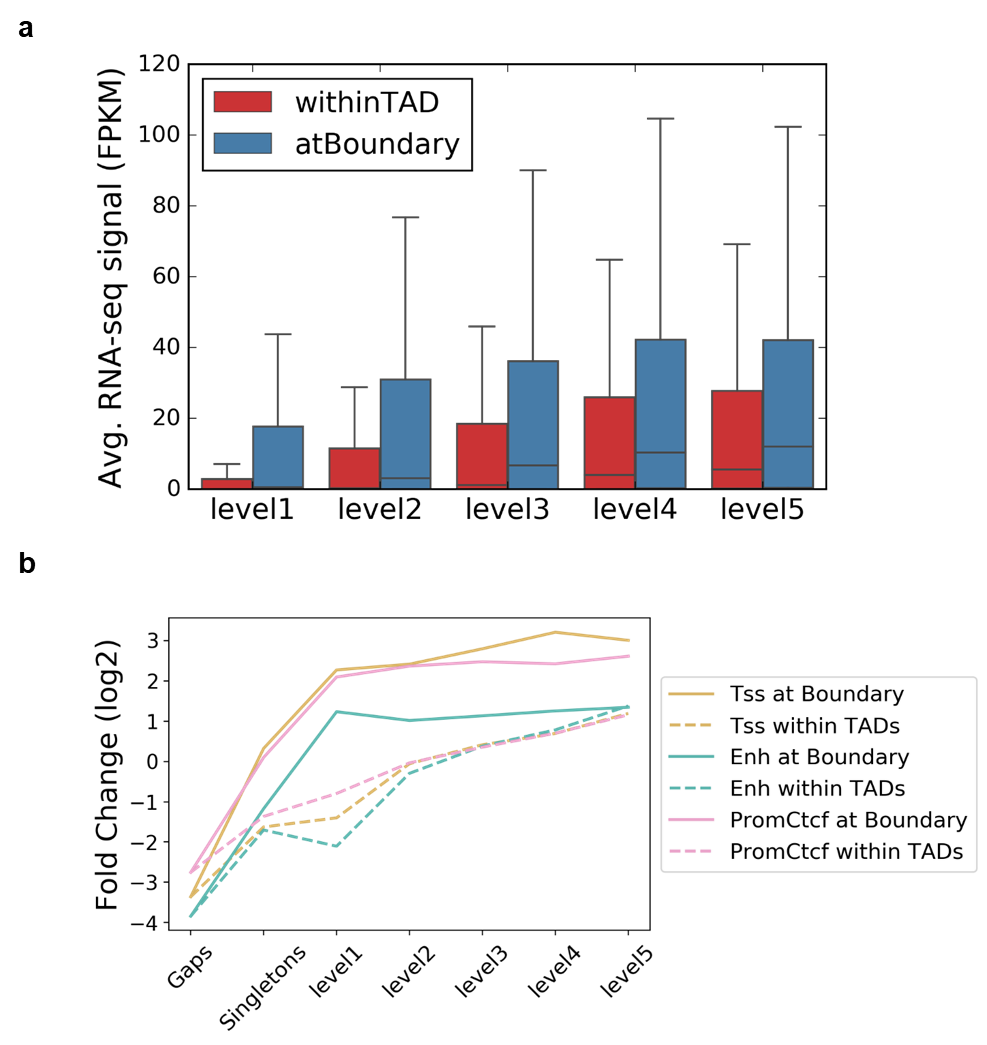 |
| --- |

**Figure S5** | **Comparison between boundaries and inside TADs** **a,** Distribution of RNA-seq signal (FPKM) at the boundaries (blue) and within TADs (red) **b,** Enrichment of active epigenetic states at the TAD boundaries (solid line) versus inside TADs (dashed line). Y-axis denotes fold enrichment of three active epigenetic states (Tss, Enh and PromCtcf). X-axis denotes the boundaries and TADs at different levels.

| 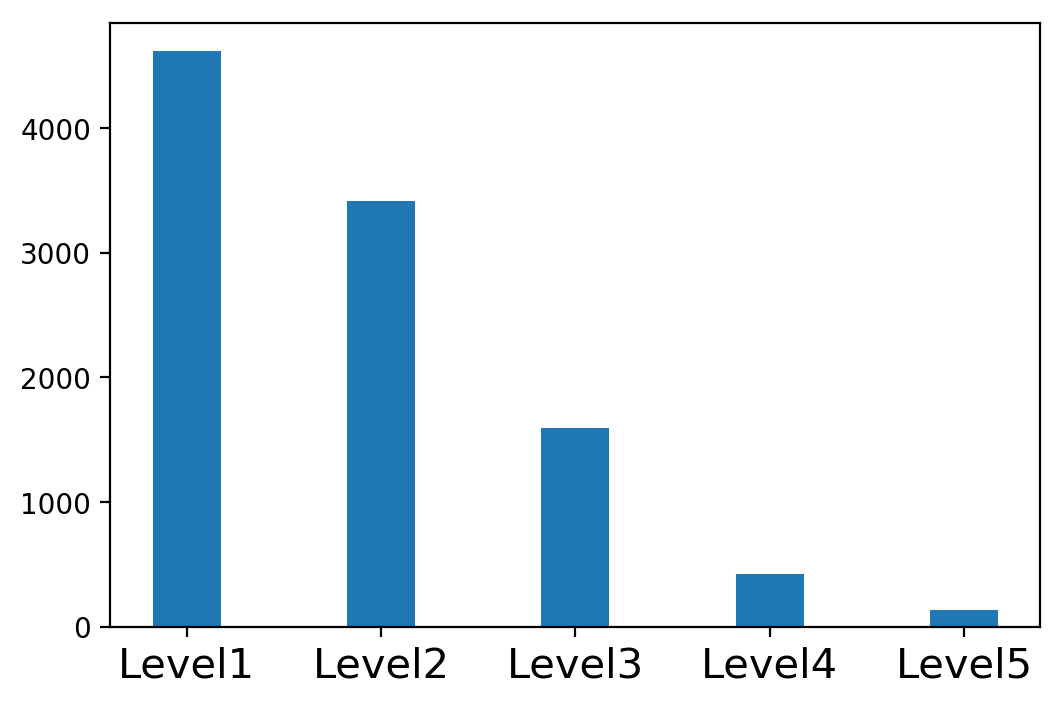 |
| --- |

**Figure S6** | **Distribution of the levels of TAD boundaries.**

| 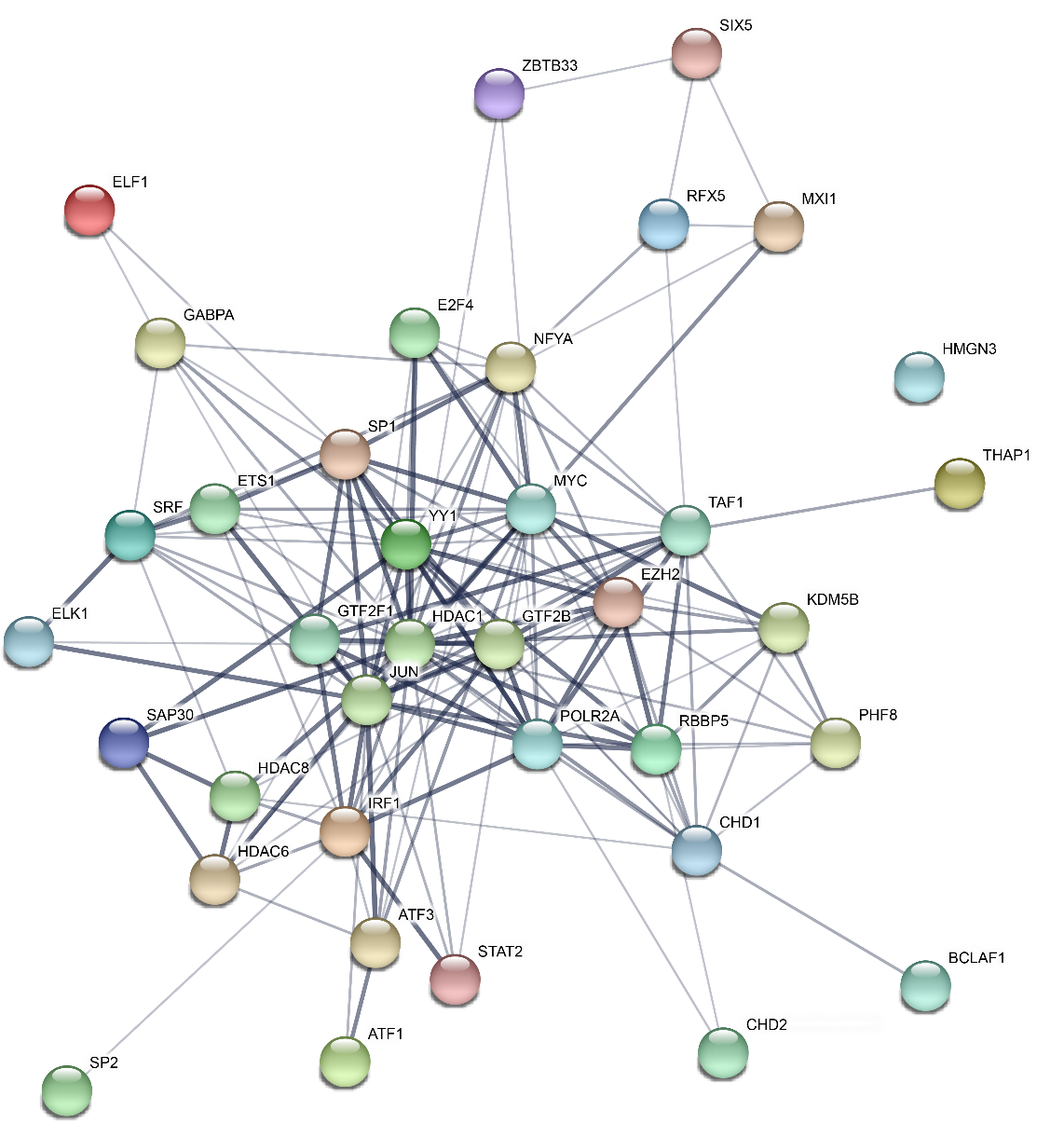 |
| --- |

**Figure S7 | Protein-protein interaction network of hub-boundary-enriched TFs from STRING database.** Each node denotes a TF that are at least 2-fold enriched at hub-boundary over level1 boundary (n=37). Each edge denotes the interaction potential between two TFs, with thicker edges corresponding to higher interaction confidence. Interaction data was downloaded from STRING database (https://string-db.org/)

| 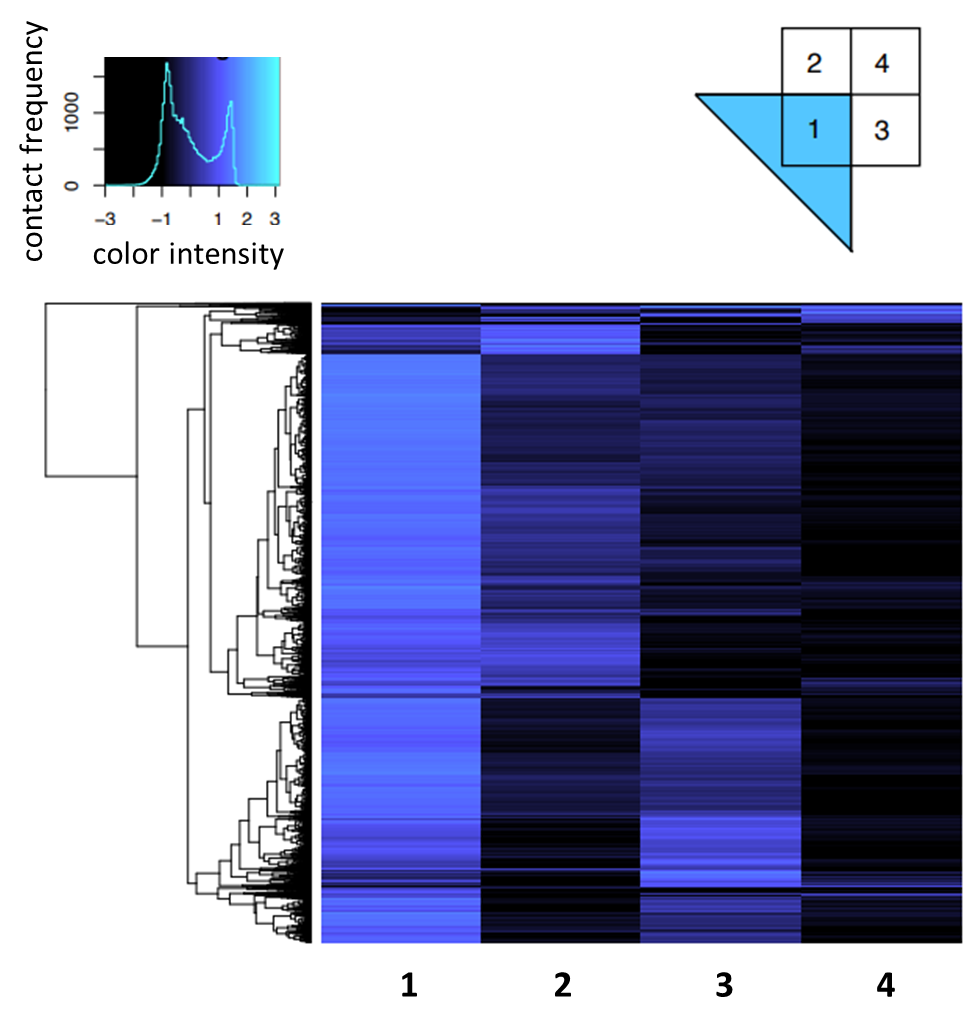 |
| --- |

**Figure S8 | Contact frequency is unbalanced between the two sides of hierarchical TAD corners.** The regions around TAD corners are segregated into four quadrants (1-4 on the top right figure). We then averaged contact frequency of each TAD corner by quadrants. As shown in the heatmap, the majority quadrant 2 and 3 shows unequal average contact frequencies, suggesting that the inner TADs tend to be formed on one side of the outer TADs, rather than on both sides. Quadrant 1 has the highest average contact frequency because it is within TADs.

| 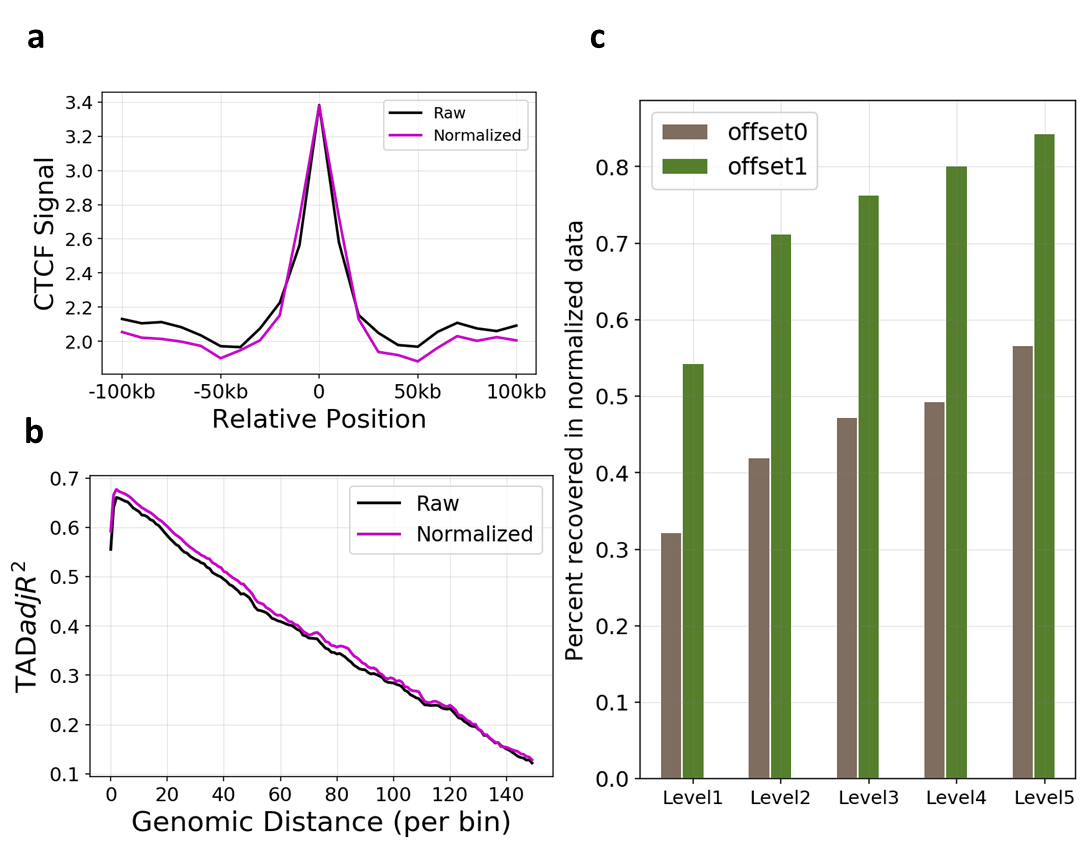 |
| --- |

**Figure S9 | Comparison of OnTAD results between raw Hi-C and normalized Hi-C in GM12878 (10kb).** **a**, Enrichment of CTCF signal at identified TAD boundaries and surrounding regions (+/- 10 bins) in raw Hi-C matrix and normalized Hi-C matrix. Y-axis: The average ChIP-Seq signal. **b**, TAD-adjR^2^ of OnTAD results at difference genomic distance in raw Hi-C matrix and normalized Hi-C matrix. The results on normalized data show a slightly higher enrichment of CTCF at boundary and a higher TAD-adjR^2^. The normalized Hi-C matrices were generated by Knight-Ruiz balancing [40] method. **c**, The proportion of boundaries identified in raw data that are recovered in normalized data. Grey: exact match; Green: one bin offset allowed when matching the boundaries identified in raw and normalized data. Half of the boundaries identified in the raw data precisely match with the boundaries identified in the normalized data. If we allow one bin offset when matching the locations of the boundaries, over 71% of the high-level TAD boundaries (level 2+) are matched between the results from raw data and normalized data.

| 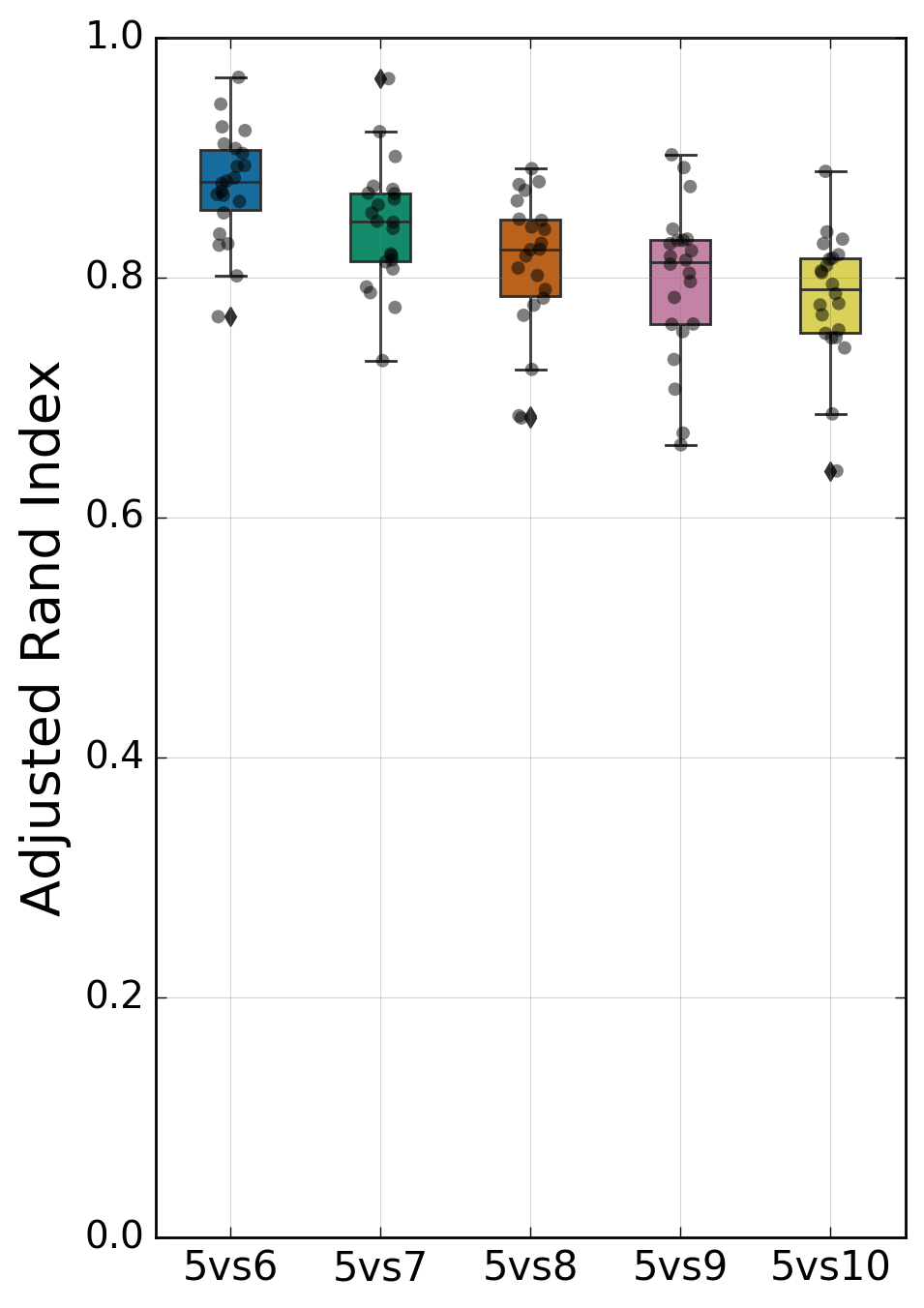 |
| --- |

**Figure S10| Similarity between the (sub)TADs identified at Lsize = 5 and at other Lsizes.**

| 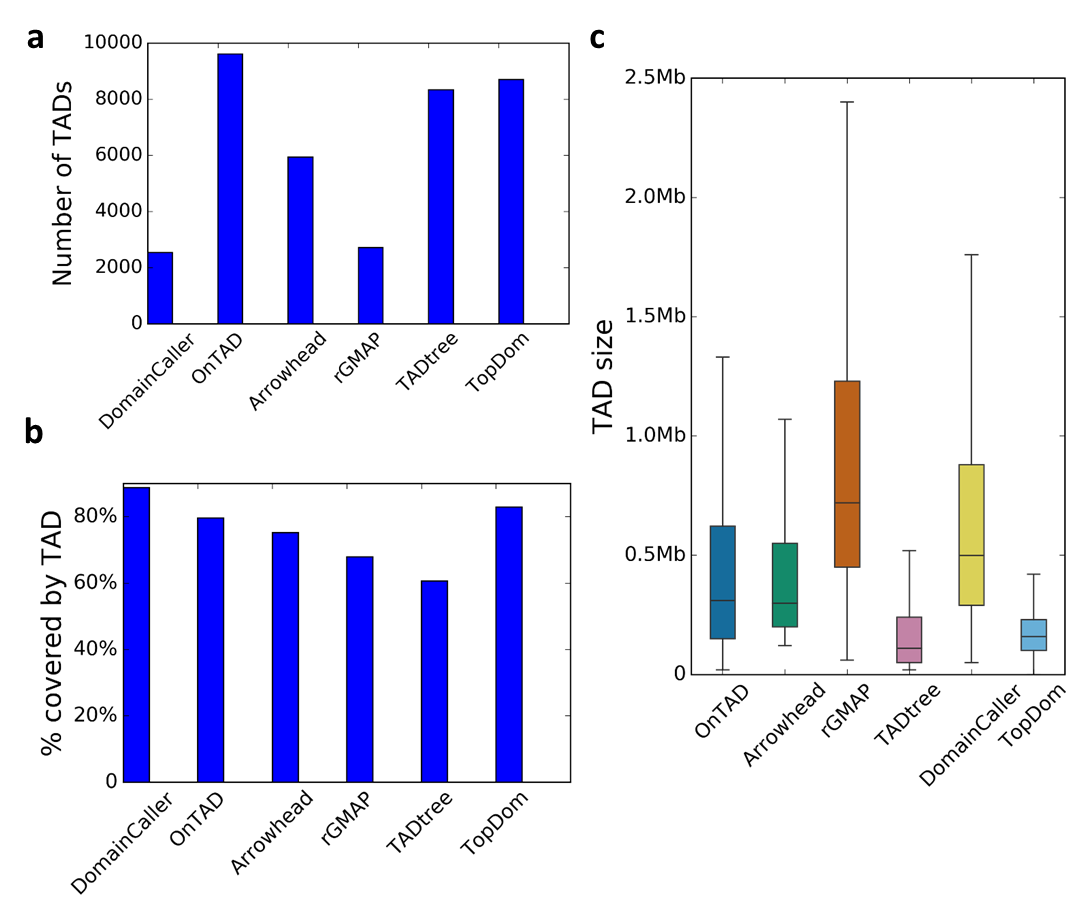 |
| --- |

**Figure S11 | Property of TAD calling results from multiple methods. a,** Number of TADs called by different methods. **b,** Percentage of genome covered by TADs. **c,** Size of TADs called by different methods. Note: TADtree has difficulty for chr1-3 at 10Kb resolution. Thus these three chromosomes were excluded for all TAD callers in all comparisons.

**Table S1: Comparison of running time (seconds) for different methods on high resolution Hi-C data (GM12878 10Kb).**

|  | OnTAD | Arrowhead | rGMAP | DIcaller | TopDom | TADtree* |
| --- | --- | --- | --- | --- | --- | --- |
| chr1 | 83 | 225 | 2902 | 1669 | 240 | - |
| chr2 | 73 | 234 | 1563 | 1463 | 225 | - |
| chr3 | 59 | 174 | 1215 | 1018 | 141 | - |
| chr4 | 55 | 168 | 1139 | 1023 | 135 | 56742 |
| chr5 | 46 | 161 | 1015 | 1111 | 143 | 44751 |
| chr6 | 41 | 153 | 1069 | 1057 | 108 | 26086 |
| chr7 | 36 | 105 | 832 | 925 | 90 | 14708 |
| chr8 | 32 | 98 | 789 | 739 | 78 | 9270 |
| chr9 | 30 | 91 | 2085 | 658 | 75 | 8997 |
| chr10 | 27 | 94 | 721 | 851 | 67 | 8365 |
| chr11 | 28 | 91 | 704 | 839 | 68 | 8368 |
| chr12 | 27 | 87 | 729 | 765 | 66 | 8166 |
| chr13 | 22 | 82 | 443 | 578 | 50 | 6874 |
| chr14 | 17 | 74 | 403 | 469 | 43 | 6584 |
| chr15 | 16 | 72 | 782 | 328 | 40 | 5984 |
| chr16 | 13 | 63 | 387 | 463 | 32 | 5363 |
| chr17 | 12 | 67 | 323 | 444 | 29 | 4119 |
| chr18 | 11 | 57 | 337 | 429 | 27 | 3926 |
| chr19 | 8 | 38 | 222 | 246 | 16 | 2896 |
| chr20 | 8 | 40 | 487 | 304 | 19 | 3065 |
| chr21 | 5 | 30 | 153 | 279 | 10 | 2417 |
| chr22 | 6 | 32 | 190 | 183 | 12 | 2559 |
| Total | 655 | 2236 | 18490 | 15841 | 1714 | 172498 |

***TADtree failed to finish running on chr 1-3 in 120hrs on the high performance computing cluster (Xeon E5-2680CPU and 72Gb RAM).**

**Table S2: Number of TADs on each side of a boundary that share this boundary (GM12878 10Kb).**

| Num of TADs on left\right | 0 | 1 | 2 | 3 | 4 | 5 |
| --- | --- | --- | --- | --- | --- | --- |
| 0 | 0 | 1297 | 378 | 110 | 23 | 9 |
| 1 | 1209 | 2108 | 1060 | 375 | 77 | 19 |
| 2 | 428 | 1023 | 524 | 214 | 80 | 27 |
| 3 | 100 | 413 | 247 | 136 | 41 | 7 |
| 4 | 22 | 71 | 61 | 35 | 12 | 3 |
| 5 | 7 | 24 | 17 | 17 | 5 | 1 |

| **Table S3:** **The FDR and number of TADs under each penalty value. (GM12878, average on 100 permutations)** |
| --- |
| \| Penalty (λ) \| FDR \| # of TADs \| \| --- \| --- \| --- \| \|  \|  \|  \| \| **0.0** \| 0.089 \| 15058 \| \| **0.1** \| 0.054 \| 13236 \| \| **0.2** \| 0.041 \| 11371 \| \| **0.3** \| 0.020 \| 9461 \| \| **0.4** \| 0.015 \| 7848 \| \| **0.5** \| 0.011 \| 6394 \| |
| **Table S4: The FDR and number of TADs under each penalty value. (G1E-ER4, average on 100 permutations)** |
| \| Penalty (λ) \| FDR \| # of TADs \| \| --- \| --- \| --- \| \|  \|  \|  \| \| **0.0** \| 0.083 \| 9025 \| \| **0.1** \| 0.028 \| 7586 \| \| **0.2** \| 0.017 \| 6303 \| \| **0.3** \| 0.013 \| 5155 \| \| **0.4** \| 0.005 \| 4230 \| \| **0.5** \| 0.005 \| 3383 \| |
| **Table S5: The FDR and number of TADs under each Lsize. (GM12878, average on 100 permutations)** |
| \| Lsize \| FDR \| # of TADs \| \| --- \| --- \| --- \| \|  \|  \|  \| \| **3** \| 0.058 \| 12987 \| \| **4** \| 0.070 \| 13579 \| \| **5** \| 0.055 \| 13236 \| \| **6** \| 0.053 \| 12626 \| \| **7** \| 0.037 \| 11920 \| \| **8** \| 0.036 \| 11287 \| \| **9** \| 0.038 \| 10677 \| \| **10** \| 0.038 \| 10147 \| |
